# Red Blood Cells: Tethering, Vesiculation, and Disease in Micro-Vascular Flow

**DOI:** 10.3390/diagnostics11060971

**Published:** 2021-05-27

**Authors:** Robert J. Asaro, Pedro Cabrales

**Affiliations:** 1 Department of Structural Engineering, University of California, San Diego, CA 92093, USA; 2 Department of Bioengineering, University of California, San Diego, CA 92093, USA; pcabrales@ucsd.edu

**Keywords:** vesiculation, hemolysis, adhesion

## Abstract

The red blood cell has become implicated in the progression of a range of diseases; mechanisms by which red cells are involved appear to include the transport of inflammatory species via red cell-derived vesicles. We review this role of RBCs in diseases such as diabetes mellitus, sickle cell anemia, polycythemia vera, central retinal vein occlusion, Gaucher disease, atherosclerosis, and myeloproliferative neoplasms. We propose a possibly unifying, and novel, paradigm for the inducement of RBC vesiculation during vascular flow of red cells adhered to the vascular endothelium as well as to the red pulp of the spleen. Indeed, we review the evidence for this hypothesis that links physiological conditions favoring both vesiculation and enhanced RBC adhesion and demonstrate the veracity of this hypothesis by way of a specific example occurring in splenic flow which we argue has various renderings in a wide range of vascular flows, in particular microvascular flows. We provide a mechanistic basis for membrane loss and the formation of lysed red blood cells in the spleen that may mediate their turnover. Our detailed explanation for this example also makes clear what features of red cell *deformability* are involved in the vesiculation process and hence require quantification and a new form of quantitative indexing.

## 1. Introduction and Goals

The red blood cell has become implicated in the progression of a range of disease states; mechanisms by which red cells are involved appear to include the transport of inflammatory molecular species via red cell-derived vesicles [1,2,3,4,5,6,7,8,9,10,11,12,13,14,15,16,17]. Moreover, the role of enhanced red cell adhesion to the vascular endothelium has been also implicated as an element of the process by which vesicles are released [18,19,20,21,22,23,24,25,26], and thereby in the progression of a wide range of dysfunctions; these include, inter alia, polycythemia vera [12,18,19,20,21]; central retinal vein occlusion [27,28,29]; Gaucher disease [26,30]; myeloproliferative neoplasms [31]; atherosclerosis [1,2,4]; sickle cell disease [32,33,34,35]; and diabetes mellitus [11,36,37]. This range of disease states suggests that some unifying principles may exist. We attempt to describe such principles via a description of mechanisms that lead to the loss of red cell membrane, vesiculation, and even possible cell lysis using the specific example of microvascular flow within the spleen. We demonstrate how the paradigm we develop for splenic flow is applicable to a far wider range of microcirculatory flow scenarios.

In this review and commentary we present evidence that vesiculation of red blood cells that are adhered during shear flow contributes directly to the progression of a wide range of diseases and thereby provide a new unifying paradigm that may be of significant use in diagnosis. For a range of disease states we discuss molecular mechanisms that identify markers that may be used as well in diagnosis. Importantly, our paradigm provides a unifying framework for correlating the progression of a range of disease states that are discussed in Section 4.

Accordingly, and specifically, we demonstrate how erythrocyte adhesion, during, even modest shear flows, can lead to vesiculation and hence a loss of membrane area and thereby to possible cell lysis itself. This leads us to postulate a novel hypothesis that vesiculation, itself a self-protective mechanism [38,39,40], occurs throughout the microvascular and that although serves a beneficial purpose by enabling the red cell to shed dysfunctional material, may also cause the dissemination of molecular species that contribute to the progression of disease. We provide a brief review of this process as it occurs in the spleen, for example, as cells pass through the interendothelial slits of the venous sinus. We then discuss how this should occur in more generally throughout the microvascular and review published experimental evidence this.

Indeed, our paradigm is consistent with, and provides mechanistic insight into, the hypothesis of Klei et al. [41], i.e., that hemolysis in the spleen via loss of erythrocyte membrane and cell shrinkage, even under low shear flow, may mediate red cell turnover. In this, the role of adhesion molecules such as Lu/BCAM is vital [42,43] in the trapping, via adhesion in the red pulp, and we believe also in the sinuses, of the spleen. Our paradigm thereby helps explain the view put forth by Klei et al. [41] that, “thus trapping or retention of erythrocytes under shear flow forces induces hemolysis”.

Vesiculation, per se, may occur via alternative pathways [44,45,46,47,48,49,50,51,52,53,54]. For instance, pathways such as the formation of clathrin or caveolin coated vesicles exist that are rather different from that explored here are well known [55,56,57,58,59]. Vesiculation also occurs, for example, during blood storage [13,60,61,62,63,64,65,66,67], from cells subjected to ATP depletion [63,64,68], or in fact via stimulation by simple, but tailored, oscillatory fluid shear flow fields [44,69]. During blood storage Kuo et al. [70] have demonstrated how microvesicles are formed from erythrocytes by the action of membrane attack complex (MAC) without shear flows or cell lysis. However, to understand red cell flow patterns within the spleen, that is patterns of sequestration and clearance [71], it may indeed be necessary to consider the paradigm of cell adherence in shear flow and cell shedding of membrane as hypothesized by Klei et al. [41]. The processes that lead to cell hemolysis appear to Klei et al. [41] to be a precursor to erythrophagocytosis by red pulp resident macrophages. Thus the processes that lead to splenic vesiculation and lysis may be related to those that have been postulated to contribute to red cell-derived vesicles contributing to disease progression.

**Figure 1:**
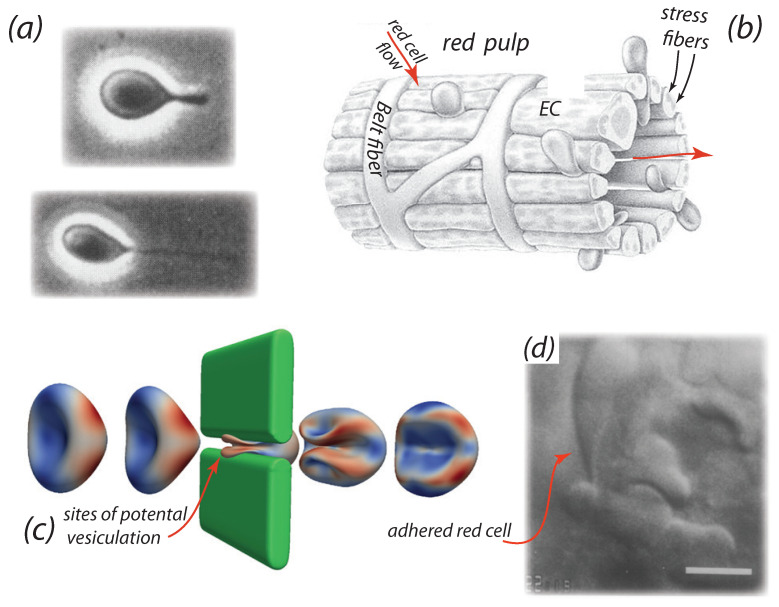
(**a**) Optical images of an RBC, adhered to a glass plate in a shear flow field forming an evagination and a tether (taken from Hochmuth et al. [72], with permission). (**b**) Schema of the splenic venous sinus and sinus wall taken from Drenckhahn and Wagner [94] (with permission). Note the stress fibers along the sides of intercellular slits whose caliber is mediated by the contractility of the myosin-actin filaments within these stress fibers [95,96,97]. Red blood cells are shown flowing through the slits. Note also the belt-like formations of the basement membrane ring fibers that abut the endothelium. Note also the “annular”, so marked in (**b**), or “ring”, fibers. (**c**) Influence of RBC initial orientation upon entering a venous splenic slit on the cell’s deformation shape. In this orientation the cell undergoes large deformations and develops an infolded region upon exiting the slit. (**d**) A snapshot of a red cell “hung up” while attempting passage thorough an IES; note this cell is not blocked but is adhered to the sinus endothelium in the IES (from MacDonald et al. [89], with permission).

Our proposed paradigm can, indeed, be applied to other forms of microcirculatory flow other than for splenic flow and throughout the vascular as noted above. We recall for example the observations of Hochmuth et al. [72] who observed evagination as well as tethers forming from RBCs while they were subjected to simple shear fluid flows while adhered to glass plates; their observations clearly fall within our paradigm’s scope. In fact they reported that “… red cells tethers steadily increased in length when the fluid shear stress is greater than 1.5 dynes/cm^2^∼0.15 Pa (see Figure 1a). As we show, this clearly falls within physiological ranges. An example of tethering of Gaucher disease RBCs [26] subject to shear flow while adhered is discussed in Section 4 to make this clear. Similar results were reported by Berk and Hochmuth [73] who observed enhanced diffusion rates for membrane proteins during tether formation that we propose may be a precursor to vesiculation; as we show below, enhanced membrane mobility of anchoring proteins tends to promote vesiculation by allowing the red cell’s skeleton to remodel under shear flow [74].

Membrane defects, especially those that involve either deficiencies in skeletal components such as spectrin or proteins associated with the sleleton-membrane connection [45,46,48,49,51], or disease states such as diabetes mellitus, or various forms of hemolytic anemia [46,47], may promote vesicultion as well as impair RBC deformability in general.

An important feature of the red cell’s structural physiology is its characteristic of deformability [75]; the importance of the property of deformability has been recognized since the earliest studies of hematology, viz. since at least 1675 by Van Leeuwenhock [76] and more recently by others in recent reviews [75,77,78,79,80,81,82,83,84]. In particular, interest in red cell fragility as may lead to hemolysis has been a focus for decades, e.g., [85,86]. Our discussion below will, however, reveal a number of vital features of a more holistic view of red cell deformability than is typically envisioned. For example, we will highlight the importance of skeletal remodeling and point to the times scales of that process as compared to overall deformation time scales; this will also highlight vital molecular effects that control such time scales as well as skeletal-membrane connectivity, and that are affected by disease states. To put a finer point on this, we recall that Knowles et al. [87] demonstrated how vesicles may be formed at the tip of a cell aspirated through a micropipette, a process that was simulated by Peng et al. [88], and that was shown by their analysis to possess quite different time scales as compared to those of any such process occurring in splenic flow, e.g., during flow through a venous slit [89]. This disparity in time scales is related to skeletal remodeling, a process that is characterized by times scales that compete with those of splenic flow; this is discussed below in detail. Skeletal remodeling involves viscous drag of transmembrane proteins such as band 3 and those associated with the actin junctional complex (JC) and thereby has its own time scales set by protein lateral mobility, as does diffusion; see [90,91,92] for specific discussion of lateral diffusion of RBC membrane proteins involved with skeletal anchorage and Lux [93] for a comprehensive review of the structure of the RBC membrane.

The plan of this commentary is as follows. Section 2 provides a brief background on the splenic venous environment, hopefully sufficient for understanding the theoretical simulations we review of red cell adhesion and tethering-leading to vesiculation. This brief account includes comparative views of RBC passage through venous slits from model simulations [44,69,98] and from in vivo observations of MacDonald et al. [89,99,100] of venous slit RBC passages in rat spleen; videos are available that provide an invaluable guide for these in vivo events and are faithfully used to ensure strict fidelity of the simulated events [74]. By doing this we illustrate some of the key mechanisms that are involved with red cell membrane-skeleton decohesion and vesiculation; the time dependence of the processes involved are emphasized. This is discussed in Section 3 where we summarize the findings of Asaro et al. [74] regarding the prospects for tethering and vesiculation of adhered red blood cells in shear flow. As the focus of that study was the detailed mathematical modeling and computational simulation of the cell deformation, here we omit that detail and only summarize the essential findings. These brief reviews are vital for understanding how and why the splenic example is connected to similar phenomena that occurs in a wide range of microcirculatory flow and how various disease states are influential in the process of vesiculation; it also helps explain how disease states are affected by RBC membrane loss, vesiculation, and possible red cell lysis. We then pass to Section 4 where we discuss more specifically how the mechanistic adhesion-vesiculation scenario relates to red cell physiology in disease states such as those that exist in, inter alia, diabetes mellitus, retinal vein occlusion (RVO), sickle cell anemia, Gaucher disease, myeloproliferative neoplasms, and atherosclerosis. The latter disease state has recently been tied to a mediating effect of RBC derived vesicles in the progression of plaque formation and its destabilization [1,4]. The proposed adhesion of erythrocytes and the formation of vesicles would augment the known effects of adhesion of leukocytes to endothelial cells of the vasculature in promoting plaque formation [23,24,25]. Finally, in Section 5 we provide a summary and suggestions for future steps that may be taken to quantify the various elements of our paradigm. These suggestions for future research should lead to more specific methods for clinical diagnosis of the severity of the disease states we discuss and possibly others.

## 2. Background on the Splenic Example

As described by Schmidt et al. [100], microcirculation through the mammalian spleen occurs via both “open” and “closed” pathways; as a consequence only a subpopulation of red blood cells that transit the spleen pass through venous slits in a given period of time. MacDonald et al. [101] have found that open circulation is via the penicillar arteries which dump into the recticular meshwork of the red pulp. Here the RBCs percolate, rather than flowing freely, through the red pulp to the venous sinuses interacting with their surroundings before re-entering the circulation and hence exist under a hemocrit as high as 78% [101]. It is in the red pulp red cells may adhere to the pulp under conditions of modest shear flow; the video in [74] provides a useful visualization of this. This should be seen in light of the findings of Klei et al. [41], viz. that adhesion in the pulp under shear flow may indeed induce membrane loss and lysis.

Erythrocytes then enter the sinuses through the interendothelial slits (IESs), as envisioned in Figure 1b, and it is during that process that adherence may again occur in what is now a quite vigorous flow field within the sinus lumen. The essential features of the sinus are illustrated in Figure 1b taken from Drenckhahn and Wagner [94]; these structural features have been also described in more recent reviews [102,103,104]. Erythrocyte slit passage has been simulated using a hierarchal multiscale molecular-continuum model [69,74,98] and, importantly, visualized via the videomicroscopy of MacDonald et al. [89] as shown in the snapshot of Figure 1d, respectively. Figure 1c is our simulation using the computational model just mentioned [69,74,98] and indicates the severe deformations that cells undergo and during such passage and identifies sites on the cell, and stages of flow, where vesiculation is most probable. MacDonald et al. [89] have provided a detailed description of the kinetics of RBC IES transit from which they had concluded that “changes in the caliber of IES (i.e., the interendothelial slits) are primarily responsible for the observed pattern of flow”; RBCs are observed to transit IESs in bursts of durations of O(10 s). In fact Chen and Weiss [105] reported that “There are no preformed apertures in the (splenic) sinus walls. Instead, slits between the sinus endothelial cells, which are otherwise closed, are widened while cells pass through them”. They went on to say, “The slits are closed except when penetrated by blood cells, platelets, or macrophages”.

We note specifically the cell shown in Figure 1d that has been “hung up” as it entered the sinus lumen; this cell remained so for approximately 10 s. Such adhesion is not uncommon and as we specifically discuss such significant times spent in this adhered state are quite relevant to the prospects of such cells forming tethers and eventually vesicles. Indeed, the question is: how does this lead to the formation of vesicles and how might such prospects be linked to disease states?

In order to appreciate the time scales for cell passage and to assess the prospects of vesiculation and related events such as the loss in bilayer lipid asymmetry, additional descriptions of cell adherence are worthwhile. The folowing discussion is brief where more detail is found in MacDonald et al. [89], Groom et al. [99], and Asaro et al. [74]; features revealed by such studies, relevant, to our hypothesis include the following.

1. The video microscopy of MacDonald et al. [89,99] shows that venous slits open in “bursts” of durations of ∼10 s; moreover adjacent slits open and close in an asynchronous manner. Mediators of this behavior may include contraction of stress fibers [95,96,97] that do so in an unsymmetrical fashion [74]
2. Additional cell passage through a given slit, that has trapped a cell “by its tail”, indeed occurs which demonstrates that slits are not simply “propped open” by a passing cell. See Figure 1d for an example. Time scales of cell trapping events were recorded by MacDonald et al. [89] to be of O(1–2 s) at least and were observed to persist for as long as 10 s in some cases.
3. Cells passing through a slit with a trapped cell in place do not, or may not, adhere to the previous and trapped cell. Note that PS exposure on the outer membrane of erythrocytes is known to promote RBC adhesion [14,15,28,106,107] and hence the occurrence of RBC adhesion may be quite RBC specific - i.e., some adhere and some do not, since some cells expose PS and some do not.
4. Trapping of cells by adhesion may then be related to the exposure of PS on its outer membrane leaflet, a feature known to promote adhesion to vascular endothelial cells [28,106,107,108] or by activation of Lu/BCAM-laminin-*α*5 adhesion [41,42]. PS exposure, which involves the loss of lipid concentration asymmetry, can provide a direct driver of vesiculation [44]. In addition, we note that Willekens et al. [109] have hypothesized that haemoglobin loss from erythrocytes can result from spleen facilitated vesiculation, perhaps we add in the manner recently proposed by Klei et al. [41].
5. Still again, the electronegativity of glycocalyx of these cells [110] may also induce adhesion. This is discussed below in brief as pertains, for example, to the effects of partial cancelation of repulsive negative charges on the glycocalyx of red and endothelial cells that, in turn, normally imparts a low friction between these cells and thereby allows for the free flow of RBCs.

## 3. Hypothesis: Vesiculation Due to RBC-EC Interaction

**Figure 2:**
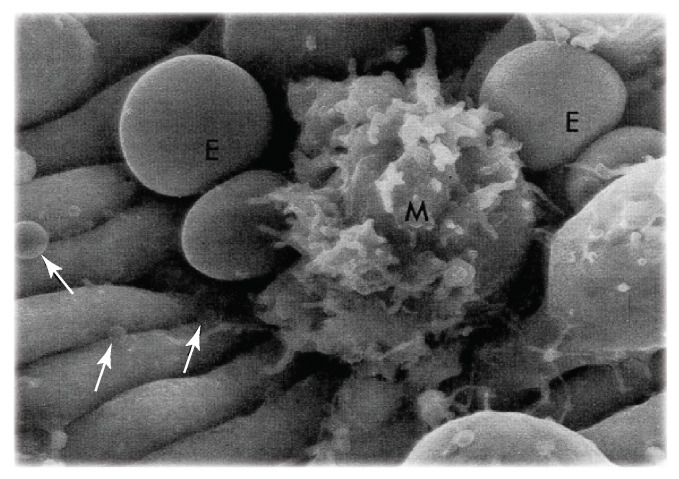
SEM image taken from Fujita [111] showing transparent vesicles (white arrows) aggregated around a human splenic sinus (with permission). The size range of those vesicles visible was in the range 150–1000 nm.

The observations and perspectives presented above provide background for quantitative for the hypothesis that: erythrocytes can be driven to vesiculate by adhesion to endothelial splenic slits via tether formation, and prompted the simulations of Asaro et al. [74]. However, their analysis and simulations also suggested that such events observed in splenic slit passage should occur in other scenarios involving, inter alia, red cell-endothelial cell interaction within the vascular; the observations of Hochmuth et al. [72], shown in Figure 1a, appear to support that notion. In particular, shear forces that exist in the red pulp of the spleen during cell adhesion may induce similar effects and hence the splenic IES passage case may be viewed as a detailed prototype of such events that lead to red cell membrane loss as hypothesized by Klei et al. [41]; indeed the SEM images, such as shown in Figure 2, of Fujita [111] revealing what were described as vesciles and red cell fragments located just outside a human sinus provide evidence for what they propose are precursors to red cell clearance.

### 3.1. Simulation Results: Cell Adhesion and Tethering

Red cell adhesion within the flow field of the rat spleen lumen was simulated in detail by Asaro et al. [74]. Their model scenario was closely tailored to the videomicroscopy observations of MacDonald et al. [89] as illustrated by the snapshots shown in Figure 1d; the imposed flow was, in fact, calibrated to match that directly recorded in the videos. Videos were included as supplementary material in Asaro et al. [74]. Here we review and summarize the essential findings so that they may provide veracity for our hypothesis as well as point to the biophysical mechanisms that lead to membrane loss, vesiculation, and hemolysis of adhered red cells.

**Figure 3:**
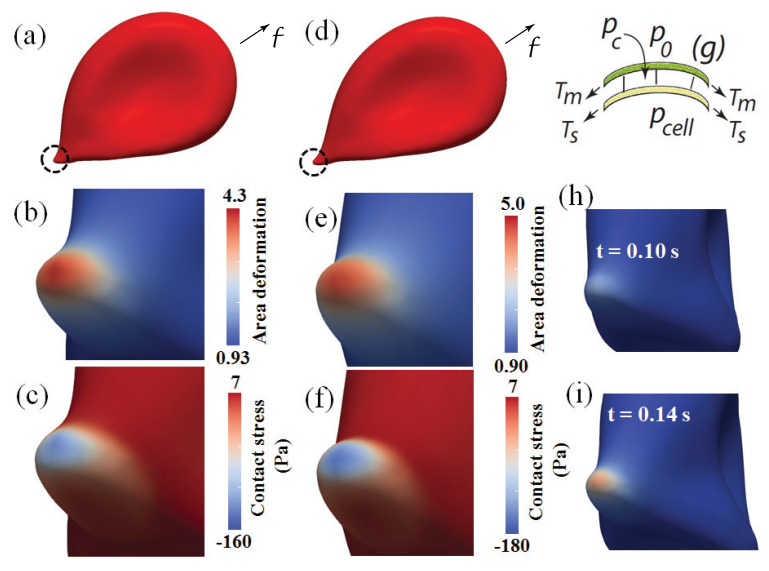
(**a**,**d**) Side views of entire cell adhered to endothelial slit walls under shear stress of τ=0.072 Pa and τ=0.15 Pa, respectively; note encircled “tips” at the cell’s adhered zone. (**b**,**e**) Contours of skeletal areal deformation for the two levels of shear stress as in (**a**,**d**). (**c**,**f**) Show contours of contact stress (pressure) defined in the text and via (**g**) that is used as a guide. (**h**,**i**) Show two snapshots of skeletal areal deformation at the times indicated for the case of τ=0.072 Pa ($\dot{\gamma }=12\;s^{-1}$); note that (**c**) shows the snapshot of areal deformation at saturation for this case.

The model used to perform these simulations accounts for full solid–fluid interactions and incorporates a molecular based thermally activated model for the spectrin-actin skeleton of the red cell and its attachments to the transmembrane protein anchors within the membrane; most particularly our model accounts for the lateral mobility of the membrane proteins and thereby for the restructuring of the skeleton that may lead to a loss in adherence of the skeleton, a factor that has quite important implications for the mechanisms of membrane loss via processes such as, inter alia, vesiculation or evagination. Details of this model can found in a number of publications, e.g., in [44,69,74]. Figure 3g illustrates the feature of our multiscale model that incorporates a fluid-like plasma membrane attached to the spectrin-actin skeleton via protein anchorages. During cell aging and/or due to disease or biochemically induced damage, such as with the presence of reactive oxygen species (ROS), the density or integrity of such attachments may be disrupted which promotes skeleton-membrane separation. Thus the goals of the simulations include assessments of the forces between the membrane and skeleton and the reductions in density of the protein attachments that are promoted by cell adherence within shear flow.

#### 3.1.1. Overall Cell Deformation and Forces

Figure 3a–f,h,i show snapshot views of cells adhered to the endothelial slit just after it had entered the sinus lumen after slit passage after a sort time of O(0.04–0.05 s); the snapshots in Figure 3a,d show the initial formation of a “tail” as evident on the left side of the cell which is upstream. The cells in Figure 3a–f,h,i were subjected to shear stresses of τ=0.072and0.15 Pa, respectively; we note that τ=0.15 Pa was the value used in the evagination/tethering experiments of Hochmuth et al. [72]. Skeletal remodeling, that is related to skeletal areal deformation—an areal expansion in this case—occurs as described by Asaro et al. and is most extensive [69,74,98] at tip areas that are encircled with dotted lines, again as shown in Figure 3a,d. The areas of high curvature such as in Figure 3b,c,e,f resemble, in terms intensity of deformation, those described as having gone through an infolding pattern that can occur at the downstream end of a cell that has passed through an endothelial slit and as is indicated to be a most likely site for vesiculation in Figure 1c. Moreover, the diameters of the evaginated bulges in these areas are on the order of 200 nm at the stage of deformation shown. Total pulling forces, depicted as *f* in Figure 3a,d, were f≈20 pN for the case where τ=0.072 Pa and f≈35 pN for τ=0.15 Pa and are quite consistent with those measured in reported tethering experiments [72,112,113,114,115,116]. Hence taken together, these observation suggest a strong prospect for tether or an evagination to form; yet this prospect also depends on the extent of skeletal remodeling discussed next.

#### 3.1.2. Skeletal Remodeling via Area Deformation: Time Scaling

As the skeleton’s area is increased, it naturally follows that the net areal density of skeleton-membrane anchorages decreases; skeletal area change is shown in Figure 3b,e for two levels of shear flow stress. Areal deformations of 4.3 and 5, as reported in those figures essentially correspond to factors of 1/4.3 and 1/5, or ∼77% or 80% reductions in the density of anchorage points. This quite significant reduction suggests, if not anticipates, de-cohesion of the skeleton-membrane interface, a known precursor to vesiculation. As skeletal remodeling requires the forced viscous motion of transmembrane anchor proteins, the force dependent time scale of this important as discussed by Asaro et al. [74] but here it should be noted that for cells with undamaged membrane-skeleton connections, these times are of O(1–3 s); for aged or disease RBCs these times will be significantly reduced due to, inter alia, ROS induced loss of such connections and enhanced protein mobility [74].

#### 3.1.3. Contact Pressure and Stress

The skeletal-membrane contact pressure, $p_{c}$, and its inverse contact stress, $\sigma_{c}=-p_{c}$, are depicted in Figure 3g; $p_{c}$ is by Laplace’s law $p_{c}=2\left(T_{m}-T_{s}\right)/r_{c}$ where $T_{m}$ and $T_{s}$ are the areal tensions in the membrane and skeleton, respectively and $r_{c}$ is their mean radius of curvature. A positive contact stress, $\sigma_{c}>0$ is essentially a normal stress tending to separate the membrane and skeleton. From Figure 3c,f it is seen that as the curvature increases in the adhesive zone the contact pressure becomes $p_{c}∼160$ Pa and $p_{c}∼180$ Pa for shear flow stresses of τ=0.072 Pa and τ=0.15 Pa, respectively. It may be recalled from Section 1 that Peng et al. [88] showed that during micropipette flow skeletal area expansions reached levels of O(3.3); in terms of skeletal density they found $\rho /\rho_{0}∼0.3$. Moreover, they found this accompanied by contact pressures of $p_{c}∼-125$ Pa. The Peng et al. [88] simulations very accurately described the pipette experiments of Hochmuth et al. [113] who reported membrane-skeleton dissociation followed, followed by tether formation and ejection of a vesicles. This is unsurprising, however, based on other perhaps even more directly related observations of tether formation and/or evagination reported by Hochmuth et al. [72] whose experimental conditions involving adhered red cells were quite similar to our present simulation scenario.

#### 3.1.4. Summary Assessment of Vesiculation Prospects

Based on the forecasted development of high contact stresses, and net reduction of the density of membrane-skeleton (M⇆S) connections, the simulations strongly support the hypothesis presented above. Moreover, the total predicted pulling force under physiological fluid shear stresses of f∼O(20 pN) shows consistency with known measured tether forces [112,113,114,117,118]. Additionally, we note that the contact pressure (stress) reaches levels of O(∼125 Pa) which is sufficient in itself to induce skeleton-membrane decohesion [88], independent of what is caused by skeleton-membrane disruption due to e.g., oxidative damage as assessed by Zhu et al. [98]; when such localized M↔S damage, i.e., de-cohered regions, exist tether formation and indeed vesiculation are strongly favored [44]. Indeed, the paradigm is consistent with the findings of Shibuya et al. [45] who correlated severe haemolysis of red cells within the human spleen with deficiencies in M⇆S connectivity involving spectrin, ankyrin and band 3.

## 4. RBC Adherence to Endothelial Cells: Pathology and Aging

The red blood cell has become increasingly implicated in a range of vascular diseases and disfunction; the review of Pernow et al. [119] provides useful general perspective. In particular, Pernow points to the role RBC-derived vesicles play in, for example, transferring “biological information acting locally to accumulate in blood vessels and be involved in atherosclerosis”. We discuss this below in reviewing the reports of Buttari et al. [1] and Boulanger et al. [2], and others, in describing mechanisms within our paradigm of adhesion-shear flow and vesiculation. Indeed, Boulanger et al. [2] point specifically to the research need to identify both the cellular source—where erythrocytes are a known candidate—and the in vivo spatiotemporal release and distribution of extracellular vesicles near atherosclerotic plaques; our paradigm provides a mechanistic pathway for vesicle formation that may prove valuable in these quests. However, there exist wide range of diseases such as those characterized by unexplained thrombosis, or vascular-occlusive events, that are associated with abnormal adhesion of erythrocytes to endothelial cells. The review by Colin et al. [120] is a brief, yet pointed, review in which diseases including sickle cell anemia, malaria, retinal vein occlusion, and Gaucher disease are discussed in this context; we add to these myeloproliferative neoplasms and atherosclerosis as examples discussed below.

On the other hand, however, we note in the examples below instances of perhaps unexpected RBC adhesion to endothelial cells that are part of a physiological process such as occurs during splenic vesiculation. For more specific review of red cell interactions with their cellular environment, including endothelial cells of the endothelium, see the reviews of Pretini et al. [27] and Krieglstein and Granger [23]. The roles that vesciles may play in disease progression in the following cases may follow the line of the role discussed in case #3 dealing with atherosclerosis.

**Figure 4:**
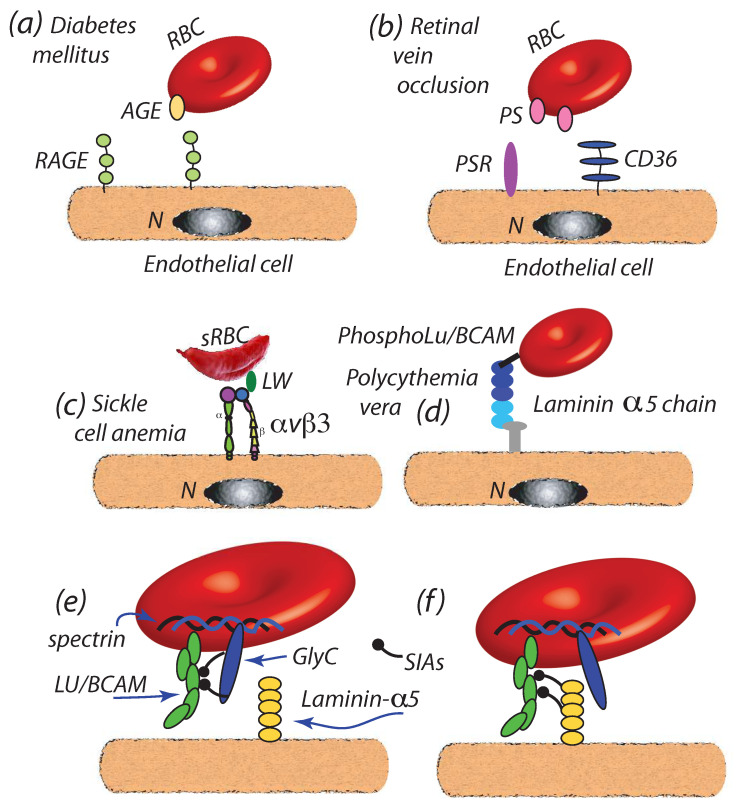
(**a**) Binding of Advanced Glycation End products (AGEs) of aged or disease RBCs to receptors for AGEs (RAGE) of the endothelium [37,131,132]. (**b**) Binding of exposed PS, due to membrane disruption or via activation of the phospholipid scramblase (PLSCR) to endothelial PS receptors (PSR) [27,107,123,135,137]. (**c**) Binding of integrins such as αvβ3 or αvβ1 to receptors such as adhesion molecule-4 (LW) on sickle cells, sRBC [33,34,35,138,139]. (**d**) Adherence of PV red cells to the endothelium by Lu/BCAM [19]. In (**a**–**d**), N is the endothelial nucleus. (**e**,**f**) Loss of sialic acid residues due to erythrocyte aging allows sialic acid binding of Lu/BCAM to laminin-α5 sialic acid residues [42] in sickle cell anemia and PV.

**Figure 5:**
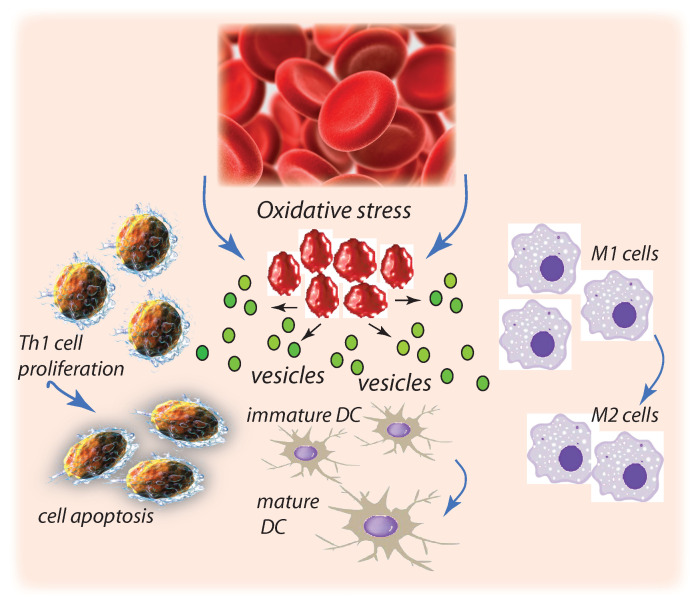
Oxidatively damaged RBCs can augment mitogen-driven T-cell proliferation and apoptosis and Th1 proinflammatory, proatherogenic cytokine response. Oxidatively stressed RBCs that do not control LPS induced DC maturation promote DC maturation thereby inciting proinflammatory Th1 cell response. Oxidatively damaged, perhaps due to storage, may polarize macrophages toward M1 pathways also promoting proinflammatory cytokine response. In short, oxidative damage of RBCs promotes those cell phenotypes that promote vesiculation and can lead to atherosclerotic progression.

1. Kucukal et al. [121,122] and Kaul [123] have reviewed the role played by red blood cell adhesion in sickle cell disease while Jambou et al. [124] have described a trogocytosis-like mechanism for the interaction of malaria infected red blood cells with human brain endothelial cells. Moreover, Wautier and Wautier [108] and Zwaal et al. [125] have reviewed research dealing focused on, and the molecular basis for, increased erythrocyte adherence to vascular endothelial cells; this included research that demonstrated enhanced adherence due to PS exposure that provided a molecular basis for enhanced RBC adherence in pathologies such as diabetes mellitus and central retinal vein occlusion [28]. Although their measurements of “adhesive strength” are of a qualitative sort, and hence precise stress levels that the adherent cells might bare unknown, it appeared that sustainable traction stress levels in excess of O(10–20 Pa) were clearly in the range [126]. Three examples are briefly discussed, followed by an additional example of a quite different type. Kucukal et al. [127], however, have preformed microfluidic testing on adhered sickle cell disease cells and using shear rates that well exceeded $\dot{\gamma }=100$ s^−1^; this would place the shear stress we quote as τ a factor of 5 above the range we demonstrated vesiculation to be forecasted at.
  (a) Diabetes mellitus: Wautier et al. [36] have reported that enhanced cell-endothelial adherence evolved in parallel with glycated hemoglobin HbA1c, wherein we note that HbA1c content is indeed found to increase in RBCs during the normal, i.e., healthy, aging process [128]; HbA1c levels have been, in fact, recommended as a diagnostic marker for diabetes mellitus [129]. The resultant Advanced Glycation End products (AGEs) so produced are ligated to the receptor for AGEs (RAGE) of endothelial cells [37,130,131,132] as depicted in Figure 4a. Full characterization of such binding is, as yet, outstanding.
    We note that increased levels of HbA1c have been positively correlated with plasma VEGF (Vascular Endothelial Growth Factor) levels and hence, that is via the influence of VGEF in angiogenesis [133]. Such effect may contribute to the progression of atherosclerosis in diabetic patients [11,134]. It is also reported that vesicle concentrations are increased with type 2 diabetes, where the role of vesicles containing inflammatory cargo, e.g., HbA1c, has also been highlighted in these recent studies [9,10,11]. Although the mechanistic cause for the VEGF-HbA1c correlation is as yet unclear, it may be suspected that HbA1c laden RBC-derived vesciles may be the trigger for unregulated VEGF expression. The general schema of such a mechanism may be as described by the red cell cross-talk depicted in Figure 5 which is discussed below, specifically in connection with atherosclerosis.
  (b) Retinal vein occlusion (RVO): retinal vein occlusion (RVO) is a common cause of permanent vision loss. Wautier and co-workers [28] reported that PS exposure was enhanced in RVO patients and correlated with enhanced cell-endothelium adhesion; the mechanism postulated was binding to PS receptors (PSR) of endothelial cells as depicted in Figure 4b; see also [27,107,135] for additional discussion of PS enhanced adhesion. When vascular leakage occurs, due to increased intraluminal pressure, this may lead to ischemia and the secretion of VEGF that may cause further leakage and retinal edema [29,136]. Hence, VEGF appears to play a significant role in RVO progression. It may be noted that enhanced PS exposure is also commonly found in other pathologies such as sickle cell anemia [123,137] wherein enhanced cell adhesion is also observed.
  (c) Sickle cell disease (SCD): recent studies show that the critical flow shear stress to detach a sickle mature erythrocyte in oxygenated is 3.9–5.5 Pa in oxygenated state and above 6.2 Pa in deoxygenated state, although multiple adhesion sites may be involved [32]. In SCD a number of proteins have been implicated in adhesion to the endothelium including integrins such as αvβ3 or αvβ1 binding to receptors such as adhesion molecule-4 (LW) and CD36 [33,34,35,138,139], as depicted in Figure 4c. It is known that adhesion in such cases can support traction stresses well in excess of 10–100 Pa and larger [126] and hence readily support binding as envisioned herein. In fact, Kaul et al. [33] found that blocking αvβ3 “may constitute a potential therapeutic approach to prevent SS RBC-endothelium interactions under flow conditions”.
  (d) *Polycythemia vera: Polycythemia vera* (PV) is a chronic disorder characterized by an increase in red cell mass resulting in hyperviscosity [19,20]. PV is also characterized by an increase in adhesiveness of red cells to the endothelium [19]; the adhesion molecule Lu/BCAM is a main player in the adhesion as depicted in Figure 4d [19,21]. It is of interest to note that Wautier et al. [19] have observed that PV cells remained adhered to human umbilical vein endothelial cells within the shear stress range 0.07 Pa ≤τ≤1.0 Pa thus demonstrating a resistance beyond the critical range of τ≈0.15 Pa found by Hochmuth et al. [72] at which tethers and evagination formed in healthy erythrocytes and the τ≈0.4 Pa that Franco et al. [26] found induced tethering in Gaucher disease RBCs (see below). Still another unaddressed line of inquiry is that, since phosphorylation of the cytoplasmic domain of Lu/BCAM is required to activate its extracellular domain, the interaction of Lu/BCAM with the erythrocyte skeleton may be quite relevant to the integrity of the M⇆S connectivity.
    Increased concentrations of microvesicles are indeed reported with PV [12,22,140] and their role in inducing thrombosis in PV noted. Tan et al. [12] specifically discuss the correlation of exposed PS on PV erythrocytes and platelets and point to the correlations of Morel et al. [141] of PS exposure and vesiculation of RBCs; biophysical mechanisms for this effect of PS have been detailed by Asaro et al. [44]. Kroll et al. [22] further point to the association of JAK2 with unregulated hematopoiatic proliferation and thrombotic risk; this is discussed further in connection with the link between erythrocute-derived microvesicles and myeloproliferative neoplasm [31].
  (e) Myeloproliferative neoplasms: Myeloproliferative neoplasms (MPNs) are hematopoietic stem cell dysfunctions that lead to over proliferation of particular cell linages. These include, inter alia, myelogenous leukemia (granulocytes); PV (RBCs); essential thrombocythemia (platelets); and neutrophilic leukemia (white cells) [142]. This extensive topic is beyond the scope of the present focused review but we note that recent findings of Poisson et al. [31] who demonstrated that vesicles derived from JAK2${}^{V617F}$ erythrocytes induce ROS and vascular endothelial constriction. In fact, Poisson et al.’s [31] Abstract illustration portrays a schema of the role of microvesicles quite reminiscent of Figure 1 of Buttari et al. [1] that we have adapted for our Figure 5. It remains to be shown if, aside from the increase in RBCs in PV, that the prospects for vesiculation in MPN RBCs is also increased. The links between those factors that lead to increased RBC adhesion and decreased membrane-skeleton connectivity and our vesiculation paradigm suggest this may well be the case.
  (f) Gaucher disease: Gaucher disease (GD) which is caused by glucocerbrosidase deficiency and is also characterized by increased RBC adherence to endothelial cells, also involves LuBCAM [26]. The study by Franco et al. [26] is particularly notable here since in their study of GD red cells, they reported that when shear flow shear stresses were raised to levels at or above τ≈0.4 Pa GD cells “exhibited frequent and elongated membrane tethers” (see their Figures 5b,c in [26]). Results for shear stresses below that range were not reported except to note that at τ∼0.1 Pa cells were reported to take on only racket-like shapes (see their Figure 5a); these were, in fact, quite similar to the simulated cell shapes of Figure 3a,d. They hypothesized that this suggested that the skeletal-membrane connectivity was altered and favored “membrane dissociation from the skeleton” [26]. Hence while adhered in a shear flow field their GD RBCs conformed to our proposed paradigm and these observations, indeed, warrant more systematic study; it is not known what the critical shear stress levels for tether formation were for GD vs. healthy red cells. Bratosin et al. [30] report that GD red blood cells do expose PS and reduced CD47, a self-recognition marker protecting against phagocytosis; this along with their abnormal shapes may mark them for early clearance, in particular in the spleen.
2. It is understood that the electronegativity of the glycocalyx of erythrocytes and endothelial cells facilitate low friction blood flow [143,144,145,146]. Accordingly, Oberieithner et al. [110] performed a series of AFM probes involving de-cohering RBCs for cultured endothelial cells in the presence of elevated $Na^{+}$, intended to reduce or neutralize net negative charge. In this they recorded increased adherence at sufficiently high $Na^{+}$ concentrations. Although of a exceedingly qualitative nature, as contact areas were not measured, nor was the geometry of “peel” profiles, they reported maximum extraction forces of O(1–1.5$\;\times \;10^{3}$ pN). Hence, even considering contact areas as large as 50–100 $m^{2}$, nominal stresses would have been greater than the range 10–50 Pa.
  We add still another comment concerning the possible role of the RBC glycocalyx regarding cell adherence, in this case cell aging. Neu et al., [147] have argued, with supporting analysis, that changes in the erythrocyte glycocalyx, in particular in its thickness, may alter the electrostatic fields between cells that affects their affinity with respect to aggregation. This view overlaps that noted above regarding the reduction of surface electronegativity that, in turn, reduces repulsion between cells and enhances adhesion. They further point out that for aged cells, which are targets for macrophage removal, that that process itself requires macrophage recognition and association, a process that requires close proximity of these cells; to accomplish close proximity macrophages and erythrocytes must overcome repulsion of a thick glycocalyx [148]. The electrostatic model they use is, in fact, quite similar to that developed by Zhu et al. [98] to describe resistance of an erythrocyte glycocalyx to compression as it attempts to squeeze through a venous slit. Hence we note the possible connections between RBC adhesion and senescence to pathologies and aging per se that affect the RBC glycocalyx, a topic that requires additional inquiry as Neu et al. indeed point out [147].
3. Atherosclerosis: red cell cross talk: atherosclerosis, a chronic progressive, multifactorial disease may be another that involves the process of erythrocyte adhesion to the endothelium within blood vessels [4,149,150,151]. The manner in which red cells may help mediate the formation and destabilization of plaque is via crosstalk between innate and adaptive immune cells, macrophages, and T lymphocytes [1,149]. For example, immune cells may be activated by various endogenous molecules that have undergone chemical or structural changes; this may include haemoglobin released from lysed RBCs or from RBC-derived vesicles [1]. Moreover, exosomes released from RBCs bind to monocytes and induce proinflammatory cytokines that boost T-cell response [150]. Red cell activity would be enhanced by oxidative stress that occurs during aging and is known to be prevalent during red cell storage [47,152]. Oxidatively stressed red cells expose PS and contain aggregated and/or oxidized HB which is released during the process of vesiculation [47]. The mechanisms we propose may indeed contribute to this process and possible mechanisms of red cell crosstalk are depicted in Figure 5 as adapted from Buttari et al. [1].
  For example, oxidized RBCs that do not control lipolysaccharide (LPS)-induced dentritic cell (DC) maturation promote DC maturation to a proinflammatory TH1 cell response [151,153]. A mechanism for this is the loss of CD47 at the erythrocyte surface due to vesiculation; CD47 appears to be critical to the role RBCs play preventing DC maturation [153]. Stored RBCs may polarize macrophages toward the M1 pathway associated with proinflammatory cytokine production. Oxidative damage promotes disruption of RBC membrane-skeleton connectivity that, in turn, induces vesiculation [44]. In addition, extracellular vesicles released by RBCs, e.g., during storage, appear capable of stimulating Th1 cells and provoking proinflammatory response by releasing cytokines [3,154].
  The role of erythrocyte-derived vessels and release of Hb in atherosclerotic lesions, and in particular within plaque neovessels [8], has been discussed with pointed reference to their effects on coagulation, inflammatory response, as well as cell adhesion per se [6,7,16,17,150]. Red cells may vesiculate more readily within the oxidative environment of atherosclerotic plaques due, in part, to the hypoxia that exists; because of hypoxic conditions there may be a switch from aerobic to anaerobic metabolism, characterized by glucose and ATP depletion [68,155], conditions that are known to promote erythrocyte vesiculation [44,64,152,156,157,158,159]. Erythrocyte-derived vesicles can then release Hb and contribute to the generation of such species as metHb and oxHb. This again suggests that vesicles produced during microvascular flow local to atherosclerotic plaques may indeed participate in plaque progression and destabilization.
  In addition, Jenny et al. [8] and Tziakas and co-workers [160,161] have hypothesized that cholesterol as well as Hb released by RBCs within atherosclerotic plaques are important contributors to plaque instability, wherein the latter refer to a mechanism involving the “breakdown of erythrocytes” releasing cholesterol; the release of Hb containing vesciles may well be part of this process although the number of vesicles would be most likely quite large to account for the cholesterol levels estimated. The possible, and probable, roles of RBC-derived vesicles in destabilizing plaques have been further reviewed by Boulanger et al. [17]. They point out the general trends of increased microvesicle levels with a range of cardiovascular risk factors including, inter alia, dyslipaemia, diabetes mellitus, hypertension, and atherosclerosis. The role of platelet derived vesicles in the development of atherosclerosis in diabetes mellitus has been discussed, in fact, for over 25 years [159].
  Taken together, the above suggests that oxidatively stressed RBCs that contain oxidized Hb, externally expose PS, and have compromised membrane-skeleton connectivity, may undergo increased endothelial cell adhesion and vesiculation via the mechanisms discussed herein. Such vesicles may then engage in cross-talk as discussed by Buttari et al. [1] thereby impacting the progression of atherosclerosis.

## 5. Discussion

### 5.1. Splenic Red Blood Cell Clearance

RBCs have a well known lifespan of about 120 days in humans [162], about half that in mice, and also about 60 days in rats; the rat model is often used as an optimal model for human red cells [163]. There has, indeed, been ample published reports on the biochemical changes, that produce markers [164], that occur with age and that are believed to lead to the clearance of senescent red cells by resident splenic red pulp macrophages (RPMs)—one such RPM is seen in the SEM image of Fujita [111] in Figure 2. However, it is curious that Gottlieb et al. [165] found that mouse senescent red cells were not phagocytosed in vitro. A possible caveat to this puzzle is that what was missing in vitro was the complement environment required to allow RPMs to “recognize” their meal. Nonetheless, Klei et al. [41] have presented a novel and provocative hypothesis that essentially states that it is not that RPMs had lost their ability to phagocytose senescent RBCs in vitro, it’s because they cannot efficiently phagocytose intact RBCs. Klei et al. [41] analyzed RPMs from human spleens and found a surprisingly low population (∼3%) of engulfed intact red cells—rather the ingested red cells appeared as ghosts. They correlated this with RBC adhesion to the red pulp via interaction between Lu/BCAM—laminin−α5 on the ligand of Lu/BCAM that is specifically activated on aged erythrocytes. They report that this adhesion interaction, under shear flow, induces RBC lysis, that appears to be a precursor to phagocytosis. Hence, a natural question is: how is this possible?

In the red pulp near the sinuses, for example, it is clear that a pressure differential exists that induces red cell flow through the pulp and then through the IESs; these pressure differentials are of the order of 1–2 mmHg (∼133–266 Pa) across the pulp-IES junction [74]. Such pressure gradients extend throughout the red pulp and induce forces on cells or order 20–50 pN, sufficient to induce tethering via the paradigm we have described. Moreover, Klei et al. [41] assessed the localization of laminin-α5 in the red pulp sinuses and suggested that it is in the sinuses, or at the sites of the IESs, that the LU/BCAM -laminin-α5 interaction occurs. That this can induce vesiculation has been made clear by the simulations of Asaro et al. [74]; how this may go further to induce lysis remains a matter for future inquiry. Figure 2 showing significant cell debris, vesicles, and spherical objects—apparently transparent—in the size range 0.2–1.5 μm in diameter makes clear that loss of cell membrane occurs. It is unclear if any RBC ghosts were imaged in the SEM studies of Fujita [111]. A few additional observations are useful.

Klei et al. [41,42] point to the role played by a loss in sialic acid residues from GlyC that enables the Lu/BCAM-laminin-α5 adhesive interaction as depicted in Figure 4e,f. Indeed, the loss in sialic acid from GlyC has been hypothesized as a mediator of erythrocyte survival in humans, rats, and rabbits for some time now [166,167,168,169,170]. Bocci [169], in fact, links the loss in sialic acid in this fashion: “… from the membrane of erythrocytes by means of a brief period of stagnation in the splenic and renal circulation in vivo by showing a much shorter half-life if extensively disialylated erythrocytes”. Banerjee et al. [171] report an unusual case of hemolytic anemia they trace to the reduction of sialylated glycoconjugates, such as glycophorin. These observations taken together with our paradigm suggest possible avenues for future study; some are outlined below.

On the other hand, Kerfoot et al. [43] also describe the binding of erythrocytes to substrates containing hyaluronic acid (HA), which is produced by endothelial cells. They identify the cell’s receptor as CD44 and demonstrate erythrocyte adhesion and cell rolling under conditions of modest shear stress in the range τ∼0.05–0.10 Pa. We note, as they also did, that this is in the range of shear flow stress that exists within the splenic red pulp and sinuses, viz. where τ∼0.07–0.15 Pa was reviewed above in Section 3.1.1 and listed within Figure 3. They, as Klei et al. [41], found that adhesion was enhanced with the loss of sialic acid from the cell’s membrane [172,173] and correlated this with cell aging; they hypothesized that such adhesion was linked with senescence and cell clearance.

### 5.2. Studies of Cell-Cell Adhesive Interaction under Shear Flow

Among the types of experimental study that may be explored to shed additional light on the prospects of red cell vesiculation and possible lysis under conditions of shear flow while adhered are the following.

1. Use of microfluidic shear flow chambers as a general set up: the studies of Hochmuth et al. [72] have already been cited; their methods are simple yet provide a useful methodology to explore the mechanisms of tether formation and possible vesiculation. It would be advisable to extend such methods to follow the pathways of hemolysis. Additional methods include those of Yang et al. [174] who studied red cell adhesion to endothelial cells via a microfluidics approach and by Alapan et al. [175] or Kucukal et al. [127] who studied adhesive effects in sickle cell disease RBCs. The latter methods provide for greater throughput and enhanced visualization. Still other microfluidic devices offer either single cell or high throughput measurement within highly controlled environments [127,176,177,178,179] and should be considered.
2. Vesiculation and lysis of senescent RBCs: the hypothesis that senescent red cells undergo hemolysis, while adhered to the red pulp, via a loss in sialic acid and under shear flow would benefit from additional mechanistic understanding. For example, questions such as, “where in the red pulp does hemolysis occur?” and “how does the bleeding of hemoglobin occur?” are relevant, or even “do membrane rafts paly a role in vesiculation leading to hemolysis [180]?” Figure 1d shows a red cell trapped as it passes through an IES; this is precisely the process analyzed by Asaro et al. [74] as reviewed by Figure 3. However, once a red cell passes through an IES into the sinus lumen, it is unclear how lysis would then occur thereafter. Hence it may be important to understand how hemolysis occurs under more modest shear flow as red cells approach, and are trapped for various times yet unknown, in more modest shear flows in the splenic red pulp. The theoretical perspective of the analysis and simulations of Asaro et al. [74] are quite relevant here as well as they provide quantitative background on details such as membrane tension and skeletal remodeling that are important for assessing pore development involved in hemoglobin loss.
  This suggests that studies involving red cells that are treated to be senescent-like as described by Klei et al. [41], and others [181,182,183,184], and that are depleted in sialic acid to induce LU/BCAM-laminin-α5 adhesion, would be advisable. The microfluidic test set-ups described above may provide for an adequate methodology.

### 5.3. Implications for Clinical Diagnosis

The paradigm discussed here involves the combined red blood cell characteristics of deformability and adhesion. Hence, to assess red cell participation in disease states as discussed herein, or the prospects for red cell clearance, both characteristics must be accounted for. This dual importance is true at both the mechanistic level how disease states are affected as well as at the clinical level with respect to the detection of disease states. Red cell deformability has long been studied as reviewed by Huisjes et al. [75], who discuss a variety of techniques for indexing deformability for possible use in diagnosis. To date, however, such techniques lack sufficient theoretical underpinning to render them truly predictive, whereas there does exist a number of empirical correlations between deformation indexes and disease to provide some guidance. An example is the use of ektacytometry, that indexes deformability as the ability of a cell to change shape [78,79,80], where correlations with sickle cell disease, β thalassemia, and hereditary spherocytosis have been attempted. On the other hand, there now exists a range of correlations of red blood cell adhesion with specific diseases coupled to the identification of the specific receptors and ligands involved. A range of these have been reviewed in Section 4 and additional reviews exist, e.g., in Wautier and Wautier [18] and Colin et al. [120]. To the cases discussed above in Section 4 we would add cells in the disease states of thalassemia [185] and malaria [186], along with cells in cold blood storage. It should be noted, however, that the specificity of cell adhesion molecules (CAMs) to various disease states is not strict since different disease states may involve the same CAMs. Nonetheless, assays of adhesion of RBCs to substrates that are functionalized with specific ligands may offer the prospect of diagnosis of vascular disease state and severity. Such assays would provide valuable signals for disease states and/or RBC senescence. High throughput, yet quantitative, spinning disc methods [187] are candidates for use for such assays as they are inexpensive and readily implemented. Moreover as reviewed above, these methods are supported by extensive theoretical analysis that allows for more quantitative interpretation.

On the other hand, single cell microfluidic studies of the type performed by Alapan et al. [175] and Kucukal et al. [122] on sickle cell disease cells offer the potential for more detailed characterization of the interplay between adhesion and deformability. As mentioned just above, these types of studies are amenable to thorough analysis and can be developed to provide a novel diagnostic tool to study related properties such as whole blood viscosity mediated by cell adhesion in disease as, for example, reported by Kucukal et al. [188]. Our proposal, however, would augment these methods by searching out specific pairs of receptors and ligands known to mediate enhanced adhesion in specific disease states.

## 6. Compliance with Ethical Standards

The authors acknowledge that this manuscript has not been published or submitted to any other journal or media. The authors, RJA and PC both contributed to the writing and planning of the manuscript. No human subjects were used in the research performed and both authors consent to the present submission. In addition, there are no conflicts of interest to report.
